# Chilblain Lupus

**DOI:** 10.31138/mjr.34.2.269

**Published:** 2023-06-30

**Authors:** Ge vivin Vinister, Rashmi Roongta, Debanjali Sinha, Arghya Chattopadhyay, Sumantro Mondal

**Affiliations:** 1 St. Sebastian Visitation Hospital, Cherthala, Kerala, India,; 2 Department of Clinical Immunology and Rheumatology, Institute of Postgraduate Medical Education and Research, Kolkata, West Bengal, India,; 3 Department of Clinical Immunology and Rheumatology, Institute of Neurosciences, Kolkata, West Bengal, India,; 4 Department of Clinical Immunology and Rheumatology, North Bengal Medical College, Darjeeling, West Bengal, India

**Keywords:** chilblain, lupus, cutaneous, acral

## CASE REPORT

**Figure 1.**
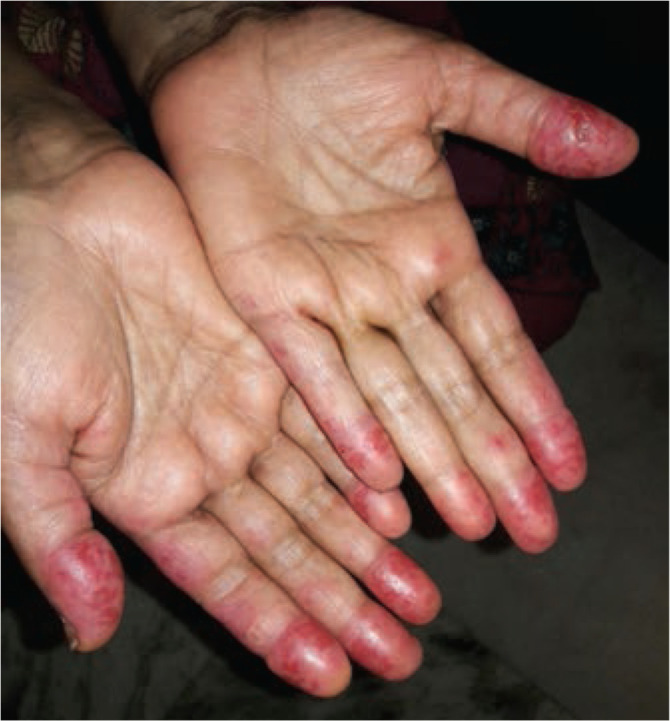
Erythematous plaque and papular lesions of chilblain lupus with ulceration over the left thumb.

A 27-year-old woman, known patient of systemic lupus erythematosus (SLE) attended our clinic in the early winters with erythematous, mildly tender plaque-like and papular cutaneous lesions involving her fingers for the last 2 weeks (**Figure 1**). She did not have a history of Raynaud’s phenomenon or symptoms compatible with involvement of other organ systems. She had been diagnosed 3 years back when she had complaints of polyarthralgia, alopecia, photosensitive malar rash, and fever. She had been on low dose steroids and hydroxychloroquine since then. Her Anti-Nuclear Antibody (ANA) test was positive at 1:320 titre, and her ANA blot showed anti-Ribonucleoprotein/Smith (RNP/Sm), anti-Sm and anti-Ro-52 antibody positivity. She had a normal complete blood count, erythrocyte sedimentation rate, liver and renal function tests. Her complement levels were low (C3: 67 mg/dL, normal 90–180mg/dL, C4: 7mg/dL, normal 10–40 mg/dL), anti- dsDNA titres were 245 IU/mL (normal <100 IU/mL) and the test for cryoglobulin was negative. She had a past history of acute cutaneous lupus erythematosus (ACLE) rash. Her skin lesions were diagnosed as chilblain lupus. Protection from cold and topical steroids were advised with which there was no discernible improvement. Tacrolimus ointment (0.1%) led to gradual improvement over the next 2 months.

## DISCUSSION

Chilblain lupus, also known as ‘Hutchinson lupus’^1^ or perniotic lupus is a rare, chronic form of cutaneous SLE characterised by mildly tender, pruritic, erythematous lesions that typically involves acral surfaces. Nose and ears are less frequently affected. Palms and soles, when involved, may develop necrosis or fissures.^2,3^ is commonly precipitated in cold weather. Impairment of microcirculation, stasis of blood and cold exacerbated vascular thrombosis are its pathogenetic factors.^3^ Anti-Ro antibodies have been associated with chilblain lupus.^4^ Histopathology shows lymphohistiocytic infiltration in superficial and deep layers of skin. The ‘Mayo Clinic Diagnostic Criteria’^5^ (**Table 1**) have been proposed for diagnosing this entity. Small vessel vasculitis is an important differential diagnosis. Lupus related vasculitis^6^ and ACLE rash^7^ are usually associated with active disease. When ACLE rash involves the hands, the interphalangeal areas of fingers are affected. Treatment of chilblain lupus includes protection from cold, topical steroids, topical calcineurin inhibitors and calcium channel blockers for relieving vasoconstriction.^2,8^ Antimalarials have a doubtful role. Systemic immunosuppression can be considered in patients who are refractory to topical treatment.^2^

**Table 1.** Proposed Mayo Clinic Diagnostic Criteria for Lupus Pernio.

**Major Criteria** Localized erythema and swelling involving acral sites and persistent for >24 h
**Minor Criteria** Onset and/or worsening in cooler months (between November and March) Histopathologic findings of skin biopsy consistent with pernio (eg, dermal oedema with superficial and deep perivascular lymphocytic infiltrate) and without findings of lupus erythematosus Response to conservative treatments (ie, warming and drying of affected areas)
**Diagnosis requires major criterion and at least one of the minor criteria to be fulfilled**
